# Effects of *Wu Qin Xi* exercise on reactive inhibition in Parkinson’s disease: A randomized controlled clinical trial

**DOI:** 10.3389/fnagi.2022.961938

**Published:** 2022-09-07

**Authors:** Zhen Wang, Yanling Pi, Xiaoyin Tan, Zhen Wang, Robert Chen, Yu Liu, Wei Guo, Jian Zhang

**Affiliations:** ^1^School of Psychology, Shanghai University of Sport, Shanghai, China; ^2^School of Exercise and Healthy Science, Xi’an Physical Education University, Xi’an, China; ^3^Institute of Medical Science, University of Toronto, Toronto, ON, Canada; ^4^Shanghai Punan Hospital of Pudong New District, Shanghai, China; ^5^Faculty of Health Sciences and Sports, Macao Polytechnic University, Macao, Macao SAR, China; ^6^School of Martial Arts, Shanghai University of Sport, Shanghai, China; ^7^Krembil Research Institute, University Health Network, Toronto, ON, Canada; ^8^Division of Neurology, Department of Medicine, University of Toronto, Toronto, ON, Canada; ^9^Key Laboratory of Exercise and Health Sciences of Ministry of Education, Shanghai University of Sport, Shanghai, China; ^10^Shanghai Yishen Health Management Co., Ltd., Shanghai, China

**Keywords:** Parkinson’s disease, reactive inhibition, proactive inhibition, physical exercise, stop signal

## Abstract

**Objective:**

Motor symptom in patients with Parkinson’s disease (PD) are related to reduced motor inhibitory ability (proactive and reactive inhibition). Although exercise has been shown to improve this ability, its effects on different levels of motor inhibition have not been determined.

**Materials and methods:**

Sixty patients with PD aged 55–75 years were allocated randomly to 24-week exercise interventions [*Wu Qin Xi* exercise (WQX) and stretching exercise (SE)]. The stop signal task and questionnaires were administered pre and post interventions. Twenty-five age-matched healthy controls were recruited to obtain reference values for inhibition.

**Results:**

Compared to healthy controls, patients with PD showed motor inhibition deficits in reactive inhibition, but not in proactive inhibition. Post-intervention, the WQX group showed significant improvement in reactive inhibition compared to the SE group. In both the WQX and SE groups, movement speed was improved post-intervention, accompanied by reduction in negative emotions, stable improvement of sleep quality, and high self-reported satisfaction levels.

**Conclusion:**

This study demonstrated that *Wu Qin Xi* exercise can improve the reactive inhibition of patients with PD. Our results provide theoretical support for the formulation of reasonable and effective exercise prescriptions for PD rehabilitation.

**Clinical trial registration:**

[http://www.chictr.org.cn], identifier [ChiCTR2000038517].

## Introduction

Patients with Parkinson’s disease (PD) have motor inhibition defects that are closely related to motor symptoms (Gauggel et al., 2004; Bokura et al., 2005; Antonelli et al., 2010; Wylie et al., 2010; Obeso et al., 2011; Bloemendaal et al., 2018; Pan et al., 2018). When facing uncertainty, reactive inhibition deficits in patients with PD result in patients tending to react prematurely or incorrectly to upcoming events, or shown more weaker proactive inhibition, being unable to adapt their motor strategy according to the context (Aron, 2011; Albares et al., 2015; Mirabella, 2021). These inhibition defects affect patients’ daily lives and quality of life (Dirnberger and Jahanshahi, 2013).

Dopaminergic medications, the mainstream PD treatment, do not alleviate motor inhibition defects (Robinson et al., 2013; Jana et al., 2020). Exercise has been used as an adjunct therapy to maintain motor function (Goodwin et al., 2008; Dibble et al., 2009; Duchesne et al., 2015), and has been shown to improve inhibitory control (Chang et al., 2012; Drollette et al., 2014). It has been suggested that the dopamine (DA) enhancing effect of exercise can partially compensate for dopaminergic deficit (Levin and Netz, 2015), related to the direct regulation of dopamine by exercise (Xiao-Xi, 2012). Studies have shown that exercise can delay the decline of executive function, likely related to improved inhibitory control (Voelcker-Rehage et al., 2011; Drollette et al., 2014).

Most exercise intervention studies conducted to improve cognitive inhibition have involved the short-term use of high-intensity exercises, such as high-intensity interval training and treadmill use (Stuckenschneider et al., 2019). Although these interventions may be effective, the training is intense, with high cardiopulmonary function and physical fitness requirements, and thus is not suitable as a long-term rehabilitation strategy in many PD patients with motor deficits. Physical and mental exercise can improve body and mind regulation in patients with PD (Kwok et al., 2019). The psychological state is related to inhibitory control; for example, such control can be enhanced by improvement in negative emotions (Pessoa, 2009). *Wu Qin Xi* (WQX), a traditional Chinese sport that integrates breathing and physical and mental regulation, which has low to medium intensity aerobic activities involving imitation of the movements and breathing of five animals (tiger, deer, bear, ape, and bird) (Guo et al., 2018). It has been shown to be an effective intervention to improve the physical and mental health in elderly adults (Zou et al., 2018). This exercise not only improves the dexterity of patients with mild to moderate PD (Wang et al., 2020), but also has been shown to reduce the risk of falls by improving balance and gait (Zhang et al., 2014). However, no conclusion has been reached regarding the effect of WQX on motor inhibition. In addition, a recent systematic review of the long-term effects of exercise revealed that most stretching training has clinically significant benefits for mobility, gait, and balance among patients with PD for the duration of its implementation (Ye et al., 2014), and stretching can reduce the shortening of flexor muscles that contributes to the abnormally flexed posture in PD patients (Corcos et al., 2013). Therefore, we also included stretch exercise (SE) in our intervention.

Two most widely used paradigms for the evaluation of motor inhibition control are the go/no-go (GNG) task (Donders, 1969) and the stop-signal task (SST) (Logan et al., 1984). GNG tasks measure the ability to suppress a potential action (action restraint), while SST measures the ability to inhibit an initiated action (action cancelation) (Mirabella, 2021). Reactive inhibition is quantified by measuring the stop-signal reaction time (SSRT), or the time it takes to inhibit an action after a stop signal is presented, and proactive inhibition is measured by determining the response delay effect. In this study, we used a modified SST (Ye et al., 2014) to examine changes in the reactive and proactive inhibition of patients with PD induced by exercise interventions.

The purpose of this study was to examine the effects of long-term low-moderate-intensity aerobic exercise (24 weeks of WQX) on motor inhibition in patients with PD, to provide insight into motor inhibition rehabilitation strategies for this patient group. We hypothesized that the WQX intervention would improve reactive and proactive inhibition in PD.

## Materials and methods

### Procedure

**FIGURE 1:**
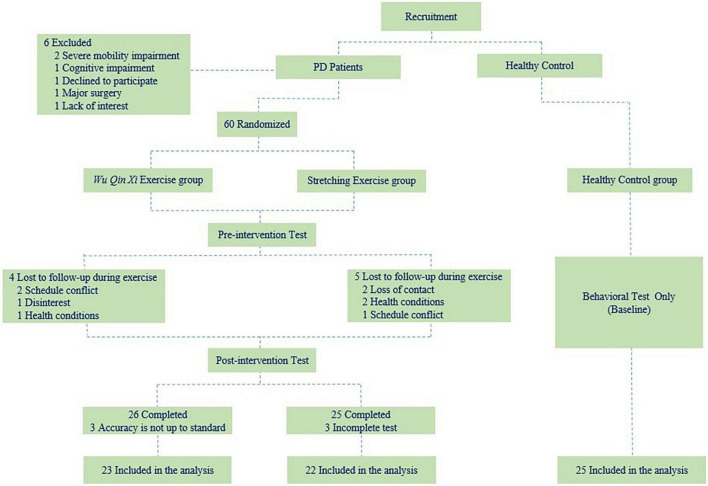
Study flow. Flow diagram of the study design.

For this prospective, single-blind, randomized controlled trial, eligible participants with PD were randomized to WQX and SE groups at a 1:1 ratio. All participants attended 24-week group-based exercise interventions at the sports laboratory of the Shanghai University of Sport (Shanghai, China). Evaluations were conducted at baseline and immediately after the intervention. All procedures were performed according to the Declaration of Helsinki and approved by the Shanghai University of Sport Ethics Committee (102772020RT107). This study has been registered in the Chinese Clinical Trial Registry (ChiCTR2000038517). All participants provided written informed consent prior to study inclusion. The study flow is illustrated in Figure 1.

### Participants

Sixty-six patients with idiopathic Parkinson’s disease were recruited through the neurology clinic of Punan Hospital, Pudong New Area, Shanghai, China, during a period of 3 months. All the patients fulfill the Movement Disorders Society Clinical Diagnostic Criteria for Parkinson’s Disease (Postuma et al., 2015). All eligible participants were right-handed (as determined by the Edinburgh Handedness Inventory) and had normal or corrected-to-normal vision. With normal cognitive function [screening for The Montreal Cognitive Assessment (MoCA)], and Hoehn & Yahr stages I–II. Additionally, those who had symptoms of impulse control disorders and were unable to stand and walk without assistive devices were excluded. The International Physical Activity Questionnaire (IPAQ) was used to collect data on their weekly physical activity. The patients in the study continued to receive medication treatment during the exercise intervention, and there were no *de novo* patients. Information on medication dosages, duration of disease, age, and behavioral symptoms were collected to ensure the information match between the two groups of PD. To explore differences in the motor inhibition of PD to age-matched healthy controls, we recruited healthy control subjects in the Yinhang community. Finally, 25 elderly people with normal or corrected right hand vision, without regular exercise and no history of neurological disease were included in study.

### Interventions

#### *Wu Qin Xi* exercise

**FIGURE 2:**
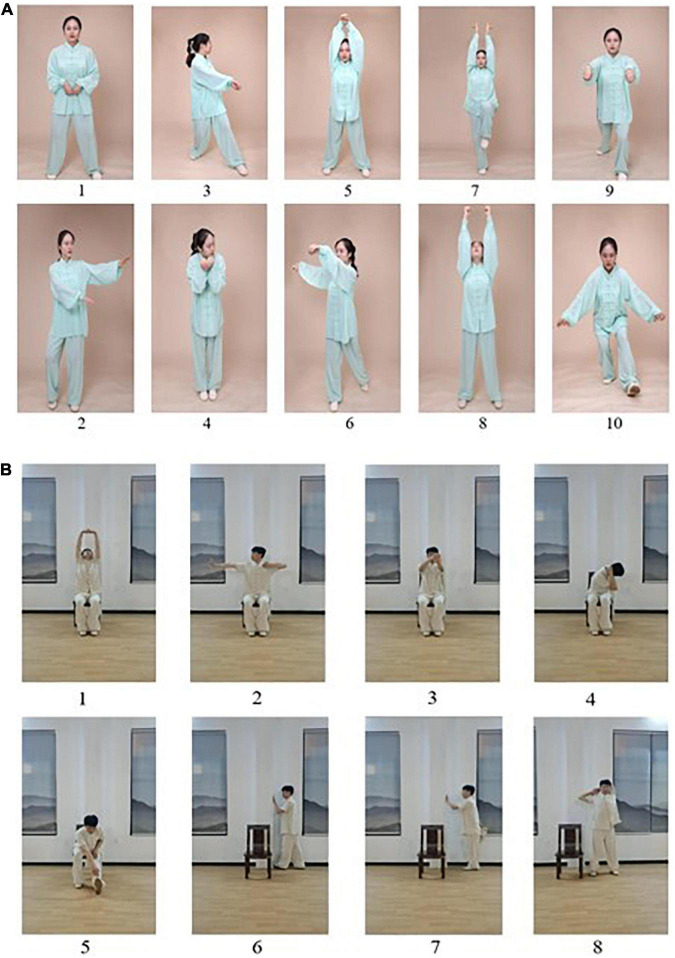
Exercise maneuvers. **(A)** *Wu Qin Xi*, a commonly practiced traditional Chinese exercise, involves full-body movements in which one imitates (with two movements each) the movements and expressions of a tiger (1, 2), a deer (3, 4), a bear (5, 6), a monkey (7, 8), and a crane (9, 10). **(B)** The stretching intervention consisted of: (1) seated stretching up and down, (2) upper-limb stretching, (3) arm stretching, (4) torso stretching, (5) lower-limb stretching, (7) front thigh stretching, and (8) shoulder stretching.

Participants in the WQX group attended a 24-week WQX exercise course with three 90-min sessions held per week. Each session consisted of 10 min warm-up followed by 60 min WQX exercise (including 10 min rest at 30 min) and 10 min cool down consisting of limb range-of-motion movements, sustained stretching, and relaxing. WQX exercise consists of full-body movements that imitate the movements and expression of a tiger, a deer, a bear, a monkey, and a bird (Figure 2A). During the intervention, participants’ performance details and heart rate (Polar-team 2 devices, Polar Electro, Finland) were recorded, in order to make real-time adjustments during the intervention based on individual condition. In the first 12 weeks, the participants were familiarized with the main components of the movements; subsequently, the practice was focused on the formal consistency of the movements and fluency of gait, posture, and balance. The participants were guided to perform the entire range of movements that felt safe to them and encouraged to practice WQX at home.

#### Stretching exercise

The SE intervention comprised supervised sessions of the same frequency, duration, and length as for the WQX intervention. It was based on an experimental exercise format and consisted of 10 min warm-up, 60 min exercise with a 10-min break at 30 min, and 10 min cooldown. The participants performed eight actions: seated (a) stretching up and down, (b) upper-limb stretching, (c) forearm extension, (d) torso stretching, and (e) lower-limb stretching and standing (f) calf stretching, (g) anterior thigh stretching, and (h) shoulder stretching (Figure 2B).

### Evaluations

All the evaluation procedure was all done in the ON state which consisted of the demographic information, questionnaires and motor inhibition tasks, totally took about 1.5–2 h per participant. Patients were assessed on mood [Hospital Anxiety and Depression Scale (HADS)], sleep [Parkinson’s Disease Sleep Scale (PDSS)], quality of life [39-item Parkinson’s Disease Questionnaire (PDQ-39)], exercise capacity [Timed Up-and-Go Test (TUGT)] and inhibition capacity (motor inhibition tasks) before and after the intervention. After the end of the questionnaires, the motor inhibition tasks were evaluated, and the order of MST and NST was counterbalanced between the subjects.

**FIGURE 3:**
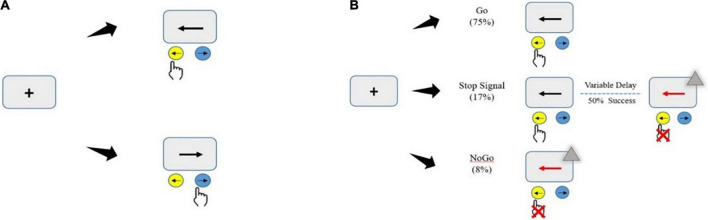
Illustration of the motor inhibition tasks. Subjects sat in a relaxed position (with the elbows, hips, and knees flexed at 90–100°) in front of a computer with a monitor in their line of sight at a distance of 75 cm. Before the test, the subjects were instructed about the experiment and completed a practice test to become familiar with the experimental task. Then, they entered the formal experiment stage and randomly completed two experimental tasks. **(A)** Never stop task (360 go-only trials). **(B)** Maybe stop task (pseudorandom mix of 75% go trials, 17% stop trials, and 8% no-go trials). The gray triangle served as the visual stop signal.

The maybe stop task (MST) was administered as a pseudorandom combination of 75% go trials, 17% stop trials, and 8% no-go trials (total, 480 trials in four blocks; Figure 3A). In go trials, subjects responded to left- and right-pointing black arrows (displayed for 1000 ms) by pressing corresponding buttons with the right index finger. In stop trials, responses were cued initially by left- and right-pointing black arrows, followed by a red arrow with a gray triangle (requiring non-response) after a stop-signal delay (SSD). The SSD (initial duration, 250 ms) was varied among trials to adjust the task difficulty using a stepwise algorithm; it was increased by 50 ms following successful non-response and decreased by 50 ms following failed non-response (Benis et al., 2014) to maintain ∼50% successful inhibition. To prevent participants from deliberately slowing their reaction times to increase the probability of correct stopping, a commonly strategy to make the stop trials easier, participants were told before the task began that the error on stop attempts had a nearly 50%, regardless of whether they delayed their response. In no-go trials, subjects were required to make no response to a red arrow with a gray triangle (displayed for 1000 ms), in a setup equivalent to a 0-ms SSD. No-go inhibition requires action selection and restraint mechanisms for the prevention of a prepotent response, whereas SSTs invoke the cancelation of an initiated response and require inhibition of motor actions. The never stop task (NST), a reaction time task including only go trials, was administered in three blocks (total, 360 trials; Figure 3B) to measure participants’ general alertness and motor speed when pressing buttons with the knowledge that no stop signal would be presented. The participants were asked to respond as quickly as possible to the stimuli presented.

### Statistical analysis

Go reaction times (RTs), SSRTs, go errors (pressing of the wrong button or missing the button), and no-go errors (pressing of a button) were taken as behavioral parameters. Trials with RTs shorter or longer than the mean ± 3 standard deviations (SDs) were considered outliers and were excluded from the analysis. Before the analysis, Shapiro–Wilk test was performed on the data distribution, all data were distributed normally.

First, analysis of variance (ANOVA) was performed to examine the baseline characteristics of the three groups. Then, ANOVA was also applied to assess differences in behavioral parameters among the three groups. The Maybe Stop and Never Stop tasks were used to differentiate between the PD groups and the healthy controls pre- and post- intervention, to determine whether there were defects in motor inhibition in PD. To examine the effects of the interventions on different types of motor inhibition, two-way repeated-measures ANOVAs were performed with the group (WQX vs. SE) serving as the between-subject variable and timepoint (baseline vs. post-intervention) serving as the within-subject variable to examine the behavioral parameters (SSRTs, RTs, context effects). The same analysis was used to compare the effects of interventions on questionnaire (e.g., PDSS, PDQ-39, TUGT, and HADS) results between the PD groups. Bonferroni correction was applied for all multiple comparisons.

To further verify the effect of the interventions on reactive inhibition, Pearson analyses were performed to assess the correlation between SSRTs and physical activity levels and UPDRS-III scores. All data were expressed as means ± SDs.

## Results

### Participant characteristics

In total, 51 PD completed the intervention. Three participants in the WQX group were excluded because they did not meet the NST accuracy performance standard of 80%. Three participants in the SE group who failed to complete all tests for health reasons were also excluded. Thus, data from 70 participants (23 in the WQX group, 22 in the SE group, and 25 healthy controls) were included in the final analysis. The demographic and clinical characteristics of participants did not differ among groups, and the results are summarized in Table 1.

**TABLE 1:** Demographic and clinical features of the participants.

	*Wu Qin Xi* Exercise group (23)	Stretching exercise group (22)	Healthy control group (25)
Age	68.83 ± 4.35	67.95 ± 4.86	66.20 ± 4.08
Male: Female	7:16	10:12	11:14
Educational level	13 ± 2.39	11.86 ± 2.64	12.84 ± 1.97
MoCA	26.70 ± 1.55	27.50 ± 1.76	26.68 ± 2.02
Levodopa (mg/day)	315 ± 134.04	347.73 ± 145.09	/
Duration of disease	6.63 ± 4.01	6.09 ± 3.85	/
Hoehn and Yahr stage	1.28 ± 0.45	1.23 ± 0.40	/

MoCA, Montreal Cognitive Assessment.

### Reactive inhibition

We first reviewed the data to explore whether the stepwise algorithm used for SSD adjustment was applicable in all study groups. The stop success probability did not differ among the three groups [*F*_(2,67)_ = 2.529, *p* = 0.087, partial η^2^ = 0.070], indicating equivalent effectiveness of this algorithm.

**FIGURE 4:**
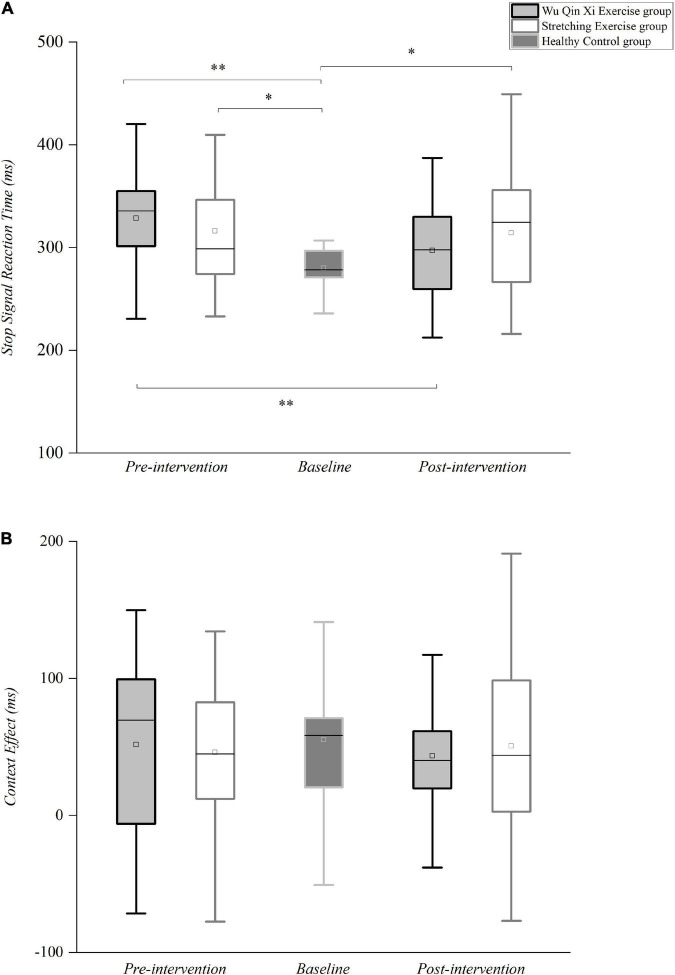
Differences in reactive and proactive motor inhibition between the healthy control and PD groups pre- and post- intervention. **(A)** Reactive inhibition. Box plot of stop-signal reaction times. **(B)** Proactive inhibition. Average context effect values. In the box plots, thick black lines represent medians, box boundaries represent lower and upper quartiles, box centers are means, and whiskers represent values 1.5 times the interquartile range. *p < 0.05, ^**^p < 0.01.

ANOVA showed that motor inhibition defects affected only the reactive inhibition in patients with PD [*F*_(2,67)_ = 6.608, *p* = 0.002, partial η^2^ = 0.165]. Bonferroni correction for multiple comparison indicated that SSRTs were significantly shorter in the healthy control group than in the WQX groups (*p* = 0.003) and SE groups (*p* = 0.038) (Figure 4A). The interventions conferred a significant main effect of time [*F*_(1,43)_ = 8.564, *p* = 0.005, partial η^2^ = 0.168], with shorter SSRT after the intervention than at baseline. However, the there was no main effect of PD groups on SSRT, but the interaction between the group and timepoint was significant [*F*_(1,43)_ = 6.976, *p* = 0.011, partial η^2^ = 0.140]. The SSRT became faster in WQX group after the intervention (297.04 ± 10.83 ms) than at baseline (328.50 ± 12.24 ms, *p* < 0.001; Figure 4A), indicating significant improvement of reactive inhibition; no such change was observed in the SE group. Compared with before intervention (*p* = 0.003), ANOVA with *post hoc* analysis revealed no significant difference in SSRTs between the WQX group and healthy controls after the intervention (*p* = 0.546), confirming that WQX effectively improved the reactive inhibition in patients with PD, to a similar level to that of healthy controls.

### Proactive inhibition

Proactive inhibition, described as motor restraint in response to contextual cues indicating increased stop-signal probability, was assessed by comparing the RTs from no-stop MST and go-only NST trials to assess the effect of context. In contrast to reactive inhibition, proactive inhibition did not differ between the PD and control groups before [*F*_(2,67)_ = 0.171, *p* = 0.843, partial η^2^ = 0.005] or after [*F*_(2,67)_ = 0.347, *p* = 0.708, partial η^2^ = 0.010] the interventions (Figure 4B). ANOVA revealed no main effect of the interventions on MST or NST RTs and no significant interaction, reflecting no significant change in the context effect (Figure 4B). Thus, patients with PD showed no obvious proactive inhibition deficit, and there was no change after 24 weeks of exercise.

### Questionnaire results

Pre- and post-intervention questionnaire scores are shown in Table 2. After the interventions, the PDQ-39 [*F*_(1,43)_ = 4.273, *p* = 0.045, partial η^2^ = 0.090], HADS [*F*_(1,43)_ = 6.231, *p* = 0.016, partial η^2^ = 0.127], PDSS [*F*_(1,43)_ = 7.729, *p* = 0.008, partial η^2^ = 0.152], UPDRS-III [*F*_(1,43)_ = 38.672, *p* < 0.001, partial η^2^ = 0.474], and TUGT [*F*_(1,43)_ = 4.452, *p* = 0.041, partial η^2^ = 0.094] showed significantly main effect of timepoint. However, no significant main effect of group or interaction between time and group was observed. The IPAQ score showed main effects of the timepoint [*F*_(1,43)_ = 11.744, *p* = 0.001, partial η^2^ = 0.215] and group [*F*_(1,43)_ = 6.251, *p* = 0.016, partial η^2^ = 0.127], and a significant interaction effect between the group and timepoint [*F*_(1,43)_ = 15.330, *p* < 0.001, partial η^2^ = 0.263]. The WQX group was more active, on average, after the intervention than at baseline (3820.09 ± 1892.09 vs. 2227.76 ± 1118.25 MET, *p* < 0.001).

**TABLE 2:** Pre- and post-intervention questionnaire scores of patients with PD.

	*Wu Qin Xi* exercise group (23)		Stretching exercise group (22)	
	Pre-intervention	Post-intervention	Pre-intervention	Post-intervention
HADS	7.87 ± 7.26	5.83 ± 4.77	10.55 ± 6.45	8.82 ± 6.85
PDSS	119.13 ± 15.89	128.57 ± 13.08	115.36 ± 16.63	119.68 ± 19.24
PDQ-39	25.78 ± 13.69	20.78 ± 14.30	30.32 ± 15.01	28.68 ± 12
UPDRS-III	20.78 ± 7.57	13.35 ± 7.33	21.18 ± 12.89	15.45 ± 7.67
IPAQ	2227.76 ± 1118.25	3820.09 ± 1892.09***	2098.64 ± 1576.15	1995.73 ± 1362.18^#^
TUGT	9.96 ± 2.17	9.54 ± 1.81	12.59 ± 5.58	11.19 ± 3.19
MoCA	26.70 ± 1.55	27.43 ± 2.11	27.5 ± 1.77	27.05 ± 2.22

PD, Parkinson’s disease; HADS, Hospital Anxiety and Depression Scale; PDSS, Parkinson’s Disease Sleep Scale; PDQ-39, 39-item Parkinson’s Disease Questionnaire; UPDRS-III, Unified Parkinson’s Disease Rating Scale part III; IPAQ, International Physical Activity Questionnaire; TUGT, Timed Up-and-Go Test; MoCA, The Montreal Cognitive Assessment.

***p < 0.001, pre-intervention vs. post-intervention; ^#^*p* < 0.05, *Wu Qin Xi* Exercise group vs. Stretching Exercise group.

### Correlation between stop-signal reaction times with physical activity levels

**FIGURE 5:**
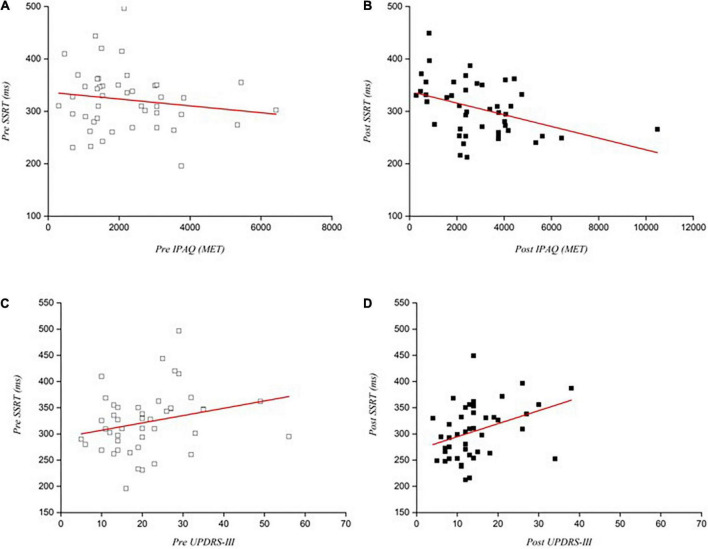
Correlations between physical activity levels and motor performance with SSRTs pre and post intervention. **(A)** Correlation between SSRTs with physical activity pre-intervention (*p* = 0.322); **(B)** Correlation between SSRTs with physical activity post-intervention (*p* = 0.006); **(C)** Correlation between SSRTs with UPDRS-III scores pre-intervention (*p* = 0.099); **(D)** Correlation between SSRTs with UPDRS-III scores post-intervention (*p* = 0.016). SSRT, Stop signal reaction time.

To examine the relationship between physical activity levels and reactive inhibition, the pre- and post-intervention SSRTs and physical activity with PD were analyzed. Pearson correlation analysis showed that SSRT and physical activity levels did not correlate pre-intervention (*r* = −0.151, *p* = 0.322; Figure 5A) but higher physical activity levels correlated with shorter SSRTs in post-intervention test in PD patients (*r* = CPS_H 0.402, *p* = 0.006; Figure 5B).

### Correlation between stop-signal reaction times with UPDRS-III scores

After 24 weeks of exercise, UPDRS-III scores showed significant reduction of the motor signs in PD patients. We analyzed the correlation between this score and reactive inhibition (SSRTs) before and after the interventions. Pearson analysis revealed no correlation pre-intervention (*r* = 0.249, *p* = 0.099; Figure 5C) but a positive correlation appeared in post-intervention between motor symptoms and SSRTs of patients with PD (*r* = 0.358, *p* = 0.016; Figure 5D).

## Discussion

We found that patients with PD had reactive, but not proactive, inhibition defects compared to healthy controls. The 24-week WQX intervention significantly improved the patients’ reactive inhibition relative to the SE intervention. Both interventions significantly improved patients’ movement speed, accompanied by improvement in negative emotions, improvement in sleep quality, and higher self-reported satisfaction levels.

### Parkinson’s disease inhibition deficits are reflected mainly in reactive inhibition control

As a bottom-up detection-driven control mechanism, reactive inhibition involves the flexible use of previous cues to resolve a present conflict (Braver et al., 2009). Our analysis of SSRTs revealed deficiencies in reactive inhibition in PD patients, which is consistent with previous studies (Obeso et al., 2013; di Caprio et al., 2020). This suggest that PD patients are slower to activate and apply the current cue information when using reaction control to resolve conflicts. The decreased inhibitory function in PD may be caused by functional changes in the frontal lobe-basal ganglia (BG) circuit caused by degeneration of dopaminergic neurons, resulting in changes in brain activity patterns, including in the prefrontal cortex (PFC) (MacDonald et al., 2000; Miller and Cohen, 2001), and changes in the functional connectivity of brain networks (Blandini et al., 2000; Fjell et al., 2017; Stuckenschneider et al., 2019). The PFC is the main cortical region activated for response inhibition during SSTs (Band and van Boxtel, 1999; Gauggel et al., 2004), and changes in its activity patterns combined with BG dysfunction (Bares et al., 2003; Farid et al., 2009) may be the basis of inhibition difficulties in patients with PD. PD patients may have difficulty sending stop signals to intercept BG output and the motor system generally when facing new situations, resulting in inhibition defects (Aron, 2011). In addition, this deficit in reactive inhibition is related to dopamine deficiency in PD. The midbrain dopamine system is responsible for gating signals to the PFC, actively maintaining task-related information (Braver and Cohen, 2000; O’Reilly et al., 2002). The gating mechanism maintains the input to PFC by means of the phasic burst of dopaminergic neuronal activities. With reduced dopaminergic input, the activation of PFC will decrease in the absence of external stimuli. Therefore, impaired reactivity control in PD may be due to reduced phase burst of DA, leading to decreased PFC activation caused by the cue, and thus interference with the continuous characterization of cue information (Xu et al., 2013).

Related to top-down cue-driven information processing, proactive inhibition is the ability to select attentional task-related cues and to actively maintain them at a subsequent time (Braver et al., 2009). Our finding of no obvious proactive inhibition deficit in patients with PD is consistent with the report of di Caprio et al. (2020). Proactive inhibition may be impaired on other clinical populations. For example, subjects with autism spectrum disorder (Mancini et al., 2018) have deficits in proactive inhibition. Moreover, proactive inhibition deficits are common in individuals with psychiatric disorders (Aron, 2011; Obeso et al., 2013), such as schizophrenia (Zandbelt et al., 2011) and bipolar disorder (Robinson et al., 2013). Although patients with PD may have anxiety and depression, these issues are more common in advanced PD (Chaudhuri and Schapira, 2009), while the participants in this study had mild to moderate PD. Additionally, changes in proactive inhibition result from the combination of cognitive processing and motor ability. Whereas the NST has one goal (going), the MST has two goals (going and stopping) (Logan et al., 1984), which increases the cognitive demands to monitor and judge different responses, thereby prolonging RTs (Verbruggen and Logan, 2009). Baseline cognitive screening revealed no cognitive impairment in our study participants. This may explain the similar levels of proactive inhibition between PD and healthy control in this study.

### 24 weeks *Wu Qin Xi* exercise can improve the reactive inhibition of patients with Parkinson’s disease

After the 24-week exercise interventions, SSRTs (reflecting reactive inhibition) were significantly shorter in the WQX group than in the SE group, with no difference for proactive inhibition. As an important part of executive control, inhibitory control is very malleable (Xu et al., 2013), which is improved significantly through training (Brevers et al., 2018). A fMRI observed changes in PFC activities related to improvements in executive function from exercise (Davis et al., 2011). Moreover, the activation of the lateral PFC (Wykes et al., 2002) can be altered by training to improve inhibition deficiencies in individuals’ schizophrenia (Edwards et al., 2010). For high-level athletes and elderly adults, systematic (long-term, repetitive) training results in improvements in inhibitory control, decision-making processes, and cue prediction when facing new situations (Brevers et al., 2018). Imaging studies have shown that training can lead to structural and morphological changes in the brain (Maguire et al., 2006; Rabipour and Raz, 2012), which including the increases in gray matter volume and the changes in white matter configuration (Wiesner et al., 2008; Scholz et al., 2009). Thus, the adult brain is plastic, even in advanced age (Xu et al., 2013). We speculate that the change in reactive inhibition in the WQX, but not SE, reflects the more comprehensive nature of WQX; whereas the SE intervention may have addressed muscle stiffness, the WQX intervention improved participants’ physical agility, flexibility, and balance. As a traditional Chinese integrated Qigong practice with physical and psychological components, WQX may have greater impact on the functional connectivity and plasticity of brain motor areas than practices such as stretching in elderly adults.

Exercise can improve motor symptoms in PD patients (Yang et al., 2014; Duchesne et al., 2015; Hampshire, 2015; Schenkman et al., 2018; Silva-Batista et al., 2018; Kwok et al., 2019; Leal et al., 2019). Training improves dopamine neurotransmission though increased dopamine release, as well as an increase in the density of postsynaptic dopamine D2 receptors in the basal ganglia (Fisher et al., 2008; Beeler et al., 2010), which may partially compensate for the loss of dopamine neurons in PD (Lees et al., 2009; Mazzoni et al., 2012; Ferrazzoli et al., 2018). Evidence showed that deficits in movement initiation in PD are related to executive and inhibitory deficits, which can be enhanced though exercise training (Albares et al., 2015). Dopamine plays a major role in the regulation of inhibitory control (Ghahremani et al., 2012). Exercise training not only enhances motor gain, but also increases dopamine release in the motor basal ganglia, which can improve inhibition in patients with PD (Smith et al., 2010; Drollette et al., 2014; Caciula et al., 2016; Fiorelli et al., 2019). These findings are also in line with our hypothesis that 24 weeks of exercise would increase the SSRTs of patients with PD, in view of WQX involves more interesting and dynamic movements, which increased patients’ interest in learning and the frequency of practice compared to stretching alone. We also found that patients in both PD groups had increased physical activity after the interventions. Participants in the WQX group exercised significantly more frequently (three times a week), and their motor symptoms decreased more than SE group. Moreover, SSRTs correlated positively with UPDRS-III scores and negatively with physical activity, which sheds light into the potential of improving the reactive inhibition in PD by exercise intervention.

In addition, we observed significant differences in PDQ-39, HADS, PDSS, and TUGT scores in the PD groups before and after the exercise interventions. Relative to baseline, patients’ positive emotion levels, motor ability, and symptom expression levels were improved after the interventions. Exercise as an adjunct therapy improves physical and mental health, enhances social skills, and promotes positive emotions. In turn, positive emotions can temporarily improve dopamine release, enhance the flexibility of working memory representation, and improve reactive control (van Wouwe et al., 2011).

## Limitation

The results from imaging studies have shown that the right IFG, DLPFC, and pre-SMA regions play important roles in reaction inhibition (Mirabella, 2014). Exercise intervention can exert a powerful influence on brain neural plasticity in PD which significantly increase cortical motor excitability and enhance serum levels of brain-derived neurotrophic factor (BDNF), in case protects against a loss of dopamine transporter binding (Mak et al., 2017). While 24-week WQX exercise can significantly improve the reactive inhibition of PD, which we concluded is fundamentally the behavioral result of optimization caused by the changes of brain plasticity, but we did not further confirm this by fMRI. In future studies, we will combine imaging and electrophysiological techniques to further explore the brain neural mechanisms regulating the changes of PD response inhibition under the effect of exercise intervention.

## Conclusion

Our study demonstrated that WQX exercise can improve the reactive inhibition in PD patients. Our results provide theoretical support for the formulation of evidence-based exercise prescriptions for PD rehabilitation.

## Data availability statement

The raw data supporting the conclusions of this article will be made available by the authors, without undue reservation.

## Ethics statement

The studies involving human participants were reviewed and approved by Shanghai University of Sport Ethics Committee (102772020RT107). The patients/participants provided their written informed consent to participate in this study. Written informed consent was obtained from the individual(s) for the publication of any potentially identifiable images or data included in this article.

## Author contributions

ZW (first author), YP, and JZ contributed to the conception and design of the study. ZW (first author), XT, WG, and ZW (fourth author) collected the data. ZW (first author) analyzed the data and wrote the manuscript. RC, YL, and JZ reviewed the manuscript. All authors contributed to the article and approved the submitted version.

## References

[B1] Albares M. Thobois S. Favre E. Broussolle E. Polo G. Domenech P. (2015). Interaction of noradrenergic pharmacological manipulation and subthalamic stimulation on movement initiation control in parkinson’s disease. *Brain Stimul.* 8 27–35. 10.1016/j.brs.2014.09.002 25284704

[B2] Antonelli F. Ray N. Strafella A. P. (2010). Imaging cognitive and behavioral symptoms in Parkinson’s disease. *Expert Rev. Neurother.* 10 1827–1838. 10.1586/ern.10.173 21091314

[B3] Aron A. (2011). From reactive to proactive and selective control: Developing a richer model for stopping inappropriate responses. *Biol. Psychiatry* 69 55–68. 10.1016/j.biopsych.2010.07.024 20932513 PMC3039712

[B4] Band G. P. H. van Boxtel G. J. M. (1999). Inhibitory motor control in stop paradigms: Review and reinterpretation of neural mechanisms. *Acta Psychol.* 101 179–211. 10.1016/s0001-6918(99)00005-0 10344185

[B5] Bares M. Kanovský P. Klajblová H. Rektor I. (2003). Intracortical inhibition and facilitation are impaired in patients with early Parkinson’s disease: A paired TMS study. *Eur. J. Neurol.* 10 385–389. 10.1046/j.1468-1331.2003.00610.x 12823490

[B6] Beeler J. A. Cao Z. F. H. Kheirbek M. A. Ding Y. Koranda J. Murakami M. (2010). Dopamine-dependent motor learning insight into levodopa’s long-duration response. *Ann. Neurol.* 67 639–647. 10.1002/ana.21947 20437561 PMC3129617

[B7] Benis D. David O. Lachaux J.-P. Seigneuret E. Krack P. Fraix V. (2014). Subthalamic nucleus activity dissociates proactive and reactive inhibition in patients with Parkinson’s disease. *Neuroimage* 91 273–281. 10.1016/j.neuroimage.2013.10.070 24368260

[B8] Blandini F. Nappi G. Tassorelli C. Martignoni E. (2000). Functional changes of the basal ganglia circuitry in Parkinson’s disease. *Prog. Neurobiol.* 62 63–88. 10.1016/S0301-0082(99)00067-2 10821982

[B9] Bloemendaal M. Froböse M. I. Wegman J. Zandbelt B. B. van de Rest O. Cools R. (2018). Neuro-cognitive effects of acute tyrosine administration on reactive and proactive response inhibition in healthy older adults. *eNeuro* 5 2355–2364. 10.1523/ENEURO.0035-17.2018 30094335 PMC6084775

[B10] Bokura H. Yamaguchi S. Kobayashi S. (2005). Event-related potentials for response inhibition in Parkinson’s disease. *Neuropsychologia* 43 967–975. 10.1016/j.neuropsychologia.2004.08.010 15716167

[B11] Braver T. S. Cohen J. D. (2000). On the control of control: The role of dopamine in regulating prefrontal function and working memory. *Atten. Perform.* 18 712–737. 10.7551/mitpress/1481.003.0044

[B12] Braver T. S. Paxton J. L. Locke H. S. Barch D. M. (2009). Flexible neural mechanisms of cognitive control within human prefrontal cortex. *Proc. Natl. Acad. Sci. U.S.A.* 106 7351–7356. 10.1073/pnas.0808187106 19380750 PMC2678630

[B13] Brevers D. Dubuisson E. Dejonghe F. Dutrieux J. Petieau M. Cheron G. (2018). Proactive and reactive motor inhibition in top athletes versus nonathletes. *Percept. Mot. Skills* 125 289–312. 10.1177/0031512517751751 29310525

[B14] Caciula M. C. Horvat M. Tomporowski P. D. Nocera J. (2016). The effects of exercise frequency on executive function in individuals with Parkinson’s disease. *Ment. Health Phys. Act.* 10 18–24. 10.1016/j.mhpa.2016.04.001

[B15] Chang Y. K. Labban J. D. Gapin J. I. Etnier J. L. (2012). The effects of acute exercise on cognitive performance: A meta-analysis. *Brain Res.* 1453 87–101. 10.1016/j.brainres.2012.02.068 22480735

[B16] Chaudhuri K. R. Schapira A. H. (2009). Non-motor symptoms of Parkinson’s disease: Dopaminergic pathophysiology and treatment. *Lancet Neurol.* 8 464–474. 10.1016/S1474-4422(09)70068-7 19375664

[B17] Corcos D. M. Robichaud J. A. David F. J. Leurgans S. E. Vaillancourt D. E. Poon C. (2013). A two-year randomized controlled trial of progressive resistance exercise for Parkinson’s disease. *Mov. Disord.* 28 1230–1240. 10.1002/mds.25380 23536417 PMC3701730

[B18] Davis C. L. Tomporowski P. D. McDowell J. E. Austin B. P. Miller P. H. Yanasak N. E. (2011). Exercise improves executive function and achievement and alters brain activation in overweight children: A randomized, Controlled Trial. *Health Psychol.* 30 91–98. 10.1037/a0021766 21299297 PMC3057917

[B19] di Caprio V. Modugno N. Mancini C. Olivola E. Mirabella G. (2020). Early-stage Parkinson’s patients show selective impairment in reactive but not proactive inhibition. *Mov. Disord.* 35 409–418. 10.1002/mds.27920 31755149

[B20] Dibble L. E. Addison O. Papa E. (2009). The effects of exercise on balance in persons with parkinson’s disease: A systematic review across the disability spectrum. *J. Neurol. Phys. Ther.* 33 14–26. 10.1097/NPT.0b013e3181990fcc 19265767

[B21] Dirnberger G. Jahanshahi M. (2013). Executive dysfunction in Parkinson’s disease: A review. *J. Neuropsychol.* 7 193–224. 10.1111/jnp.12028 24007368

[B22] Donders F. C. (1969). On the speed of mental processes. *Acta Psychol.* 30 412–431. 10.1016/0001-6918(69)90065-1 5811531

[B23] Drollette E. S. Scudder M. R. Raine L. B. Moore R. D. Saliba B. J. Pontifex M. B. (2014). Acute exercise facilitates brain function and cognition in children who need it most: An ERP study of individual differences in inhibitory control capacity. *Dev. Cogn. Neurosci.* 7 53–64. 10.1016/j.dcn.2013.11.001 24309300 PMC6987893

[B24] Duchesne C. Lungu O. Nadeau A. Robillard M. E. Boré A. Bobeuf F. (2015). Enhancing both motor and cognitive functioning in Parkinson’s disease: Aerobic exercise as a rehabilitative intervention. *Brain Cogn.* 99 68–77. 10.1016/j.bandc.2015.07.005 26263381

[B25] Edwards B. G. Barch D. M. Braver T. S. (2010). Improving prefrontal cortex function in schizophrenia throughfocused training of cognitive control. *Front. Hum. Neurosci.* 4:32. 10.3389/fnhum.2010.00032 20461148 PMC2866566

[B26] Farid K. Sibon I. Guehl D. Cuny E. Burbaud P. Allard M. (2009). Brain Dopaminergic modulation associated with executive function in Parkinson’s disease. *Mov. Disord.* 24 1962–1969. 10.1002/mds.22709 19672989

[B27] Ferrazzoli D. Ortelli P. Zivi I. Cian V. Urso E. Ghilardi M. F. (2018). Efficacy of intensive multidisciplinary rehabilitation in Parkinson’s disease: A randomised controlled study. *J. Neurol. Neurosurg. Psychiatry* 89 828–835. 10.1136/jnnp-2017-316437 29321141 PMC6204945

[B28] Fiorelli C. M. Ciolac E. G. Simieli L. Silva F. A. Fernandes B. Christofoletti G. (2019). Differential acute effect of high-intensity interval or continuous moderate exercise on cognition in individuals with Parkinson’s disease. *J. Phys. Act. Health* 16 157–164. 10.1123/jpah.2018-0189 30626260

[B29] Fisher B. E. Wu A. D. Salem G. J. Song J. Lin (Janice) C. H. Yip J. (2008). The Effect of exercise training in improving motor performance and corticomotor excitability in people with early Parkinson’s disease. *Arch. Phys. Med. Rehabil.* 89 1221–1229. 10.1016/j.apmr.2008.01.013 18534554 PMC2989816

[B30] Fjell A. M. Sneve M. H. Grydeland H. Storsve A. B. Walhovd K. B. (2017). The disconnected brain and executive function decline in aging. *Cereb. Cortex* 27 2303–2317. 10.1093/cercor/bhw082 27073220

[B31] Gauggel S. Rieger M. Rieger T.-A. (2004). Inhibition of ongoing responses in patients with Parkinson’s disease. *J. Neurol. Neurosurg. Psychiatry* 75 539–544. 10.1136/jnnp.2003.016469 15026491 PMC1739013

[B32] Ghahremani D. G. Lee B. Robertson C. L. Tabibnia G. Morgan A. T. de Shetler N. (2012). Striatal dopamine D 2/D 3 receptors mediate response inhibition and related activity in frontostriatal neural circuitry in humans. *J. Neurosci.* 32 7316–7324. 10.1523/JNEUROSCI.4284-11.2012 22623677 PMC3517177

[B33] Goodwin V. A. Richards S. H. Taylor R. S. Taylor A. H. Campbell J. L. (2008). The effectiveness of exercise interventions for people with Parkinson’s disease: A systematic review and meta-analysis. *Mov. Disord.* 23 631–640. 10.1002/mds.21922 18181210

[B34] Guo Y. Xu M. Wei Z. Hu Q. Chen Y. Yan J. (2018). Beneficial effects of qigong wuqinxi in the improvement of health condition, prevention, and treatment of chronic diseases: Evidence from a systematic review. *Evid. Based Complement. Alternat. Med.* 2018:3235950. 10.1155/2018/3235950 30473716 PMC6220394

[B35] Hampshire A. (2015). Putting the brakes on inhibitory models of frontal lobe function. *Neuroimage* 113 340–355. 10.1016/j.neuroimage.2015.03.053 25818684 PMC4441092

[B36] Jana S. Hannah R. Muralidharan V. Aron A. R. (2020). Temporal cascade of frontal, motor and muscle processes underlying human action-stopping. *Elife* 9 1–28. 10.7554/eLife.50371 32186515 PMC7159878

[B37] Kwok J. Y. Y. Kwan J. C. Y. Auyeung M. Mok V. C. T. Lau C. K. Y. Choi K. C. (2019). Effects of mindfulness yoga vs stretching and resistance training exercises on anxiety and depression for people with Parkinson disease: A randomized clinical trial. *JAMA Neurol.* 76 755–763. 10.1001/jamaneurol.2019.0534 30958514 PMC6583059

[B38] Leal L. C. P. Abrahin O. Rodrigues R. P. da Silva M. C. R. Araújo A. P. M. de Sousa E. C. (2019). Low-volume resistance training improves the functional capacity of older individuals with Parkinson’s disease. *Geriatr. Gerontol. Int.* 19 635–640. 10.1111/ggi.13682 31037806

[B39] Lees A. J. Hardy J. Revesz T. (2009). Parkinson’s disease. *Lancet* 373 2055–2066. 10.1016/S0140-6736(09)60492-X 19524782

[B40] Levin O. Netz Y. (2015). Aerobic training as a means to enhance inhibition: What’s yet to be studied? *Eur. Rev. Aging Phys. Act.* 12 1–4. 10.1186/s11556-015-0160-9 26865878 PMC4748326

[B41] Logan G. D. Cowan W. B. Davis K. A. (1984). On the ability to inhibit simple and choice reaction time responses: A model and a method. *J. Exp. Psychol. Hum. Percept. Perform.* 10 276–291. 10.1037/0096-1523.10.2.276 6232345

[B42] MacDonald A. W. Cohen J. D. Andrew Stenger V. Carter C. S. (2000). Dissociating the role of the dorsolateral prefrontal and anterior cingulate cortex in cognitive control. *Science (1979)* 288 1835–1838. 10.1126/science.288.5472.1835 10846167

[B43] Maguire E. A. Woollett K. Spiers H. J. (2006). London taxi drivers and bus drivers: A structural MRI and neuropsychological analysis. *Hippocampus* 16 1026–1031. 10.1002/hipo.20233 17024677

[B44] Mak M. K. Wong-Yu S. I. Shen X. Chung C. L. (2017). Long-term effects of exercise and physical therapy in people with Parkinson disease. *Nat. Rev. Neurol.* 13 689–703. 10.1038/nrneurol.2017.128 29027544

[B45] Mancini C. Cardona F. Baglioni V. Panunzi S. Pantano P. Suppa A. (2018). Inhibition is impaired in children with obsessive-compulsive symptoms but not in those with tics. *Mov. Disord.* 33 950–959. 10.1002/mds.27406 29781133

[B46] Mazzoni P. Shabbott B. Cortés J. C. (2012). Motor control abnormalities in Parkinson’s disease. *Cold Spring Harb. Perspect. Med.* 2 1–17. 10.1101/cshperspect.a009282 22675667 PMC3367543

[B47] Miller E. K. Cohen J. D. (2001). An integrate theory of PFC function. *Annu. Rev. Neurosci.* 24 167–202. 11283309 10.1146/annurev.neuro.24.1.167

[B48] Mirabella G. (2014). Should I stay or should I go? Conceptual underpinnings of goal-directed actions. *Front. Syst. Neurosci.* 8:206. 10.3389/fnsys.2014.00206 25404898 PMC4217496

[B49] Mirabella G. (2021). Inhibitory control and impulsive responses in neurodevelopmental disorders. *Dev. Med. Child Neurol.* 63 520–526. 10.1111/dmcn.14778 33340369

[B50] O’Reilly R. C. Noelle D. C. Braver T. S. Cohen J. D. (2002). Prefrontal cortex and dynamic categorization tasks: Representational organization and neuromodulatory control. *Cereb. Cortex* 12 246–257. 10.1093/cercor/12.3.246 11839599

[B51] Obeso I. Wilkinson L. Casabona E. Bringas M. L. Álvarez M. Álvarez L. (2011). Deficits in inhibitory control and conflict resolution on cognitive and motor tasks in Parkinson’s disease. *Exp. Brain Res.* 212 371–384. 10.1007/s00221-011-2736-6 21643718

[B52] Obeso I. Wilkinson L. Rodríguez-Oroz M. C. Obeso J. A. Jahanshahi M. (2013). Bilateral stimulation of the subthalamic nucleus has differential effects on reactive and proactive inhibition and conflict-induced slowing in Parkinson’s disease. *Exp. Brain Res.* 226 451–462. 10.1007/s00221-013-3457-9 23525560

[B53] Pan Y. Wang L. Zhang Y. Zhang C. Qiu X. Tan Y. (2018). Deep brain stimulation of the internal globus pallidus improves response initiation and proactive inhibition in patients with Parkinson’s disease. *Front. Psychol.* 9:351. 10.3389/fpsyg.2018.00351 29681869 PMC5897903

[B54] Pessoa L. (2009). How do emotion and motivation direct executive control? *Trends Cogn. Sci.* 13 160–166. 19285913 10.1016/j.tics.2009.01.006 PMC2773442

[B55] Postuma R. B. Berg D. Stern M. Poewe W. Olanow C. W. Oertel W. (2015). MDS clinical diagnostic criteria for Parkinson’s disease. *Mov. Disord.* 30 1591–1601. 10.1002/mds.26424 26474316

[B56] Rabipour S. Raz A. (2012). Training the brain: Fact and fad in cognitive and behavioral remediation. *Brain Cogn.* 79 159–179. 10.1016/j.bandc.2012.02.006 22463872

[B57] Robinson L. J. Thompson J. M. Gallagher P. Gray J. M. Young A. H. Ferrier I. N. (2013). Performance monitoring and executive control of attention in euthymic bipolar disorder: Employing the CPT-AX paradigm. *Psychiatry Res.* 210 457–464. 10.1016/j.psychres.2013.06.039 23880481

[B58] Schenkman M. Moore C. G. Kohrt W. M. Hall D. A. Delitto A. Comella C. L. (2018). Effect of high-intensity treadmill exercise on motor symptoms in patients with De Novo Parkinson disease a phase 2 randomized clinical trial. *JAMA Neurol.* 75 219–226. 10.1001/jamaneurol.2017.3517 29228079 PMC5838616

[B59] Scholz J. Klein M. C. Behrens T. E. J. Johansen-Berg H. (2009). Training induces changes in white-matter architecture. *Nat. Neurosci.* 12 1370–1371. 10.1038/nn.2412 19820707 PMC2770457

[B60] Silva-Batista C. Corcos D. M. Kanegusuku H. Piemonte M. E. P. Gobbi L. T. B. de Lima-Pardini A. C. (2018). Balance and fear of falling in subjects with Parkinson’s disease is improved after exercises with motor complexity. *Gait Posture* 61 90–97. 10.1016/j.gaitpost.2017.12.027 29310015

[B61] Smith P. J. Blumenthal J. A. Hoffman B. M. Cooper H. Strauman T. A. Welsh-Bohmer K. (2010). Aerobic exercise and neurocognitive performance: A meta-analytic review of randomized controlled trials. *Psychosom. Med.* 72 239–252. 10.1097/PSY.0b013e3181d14633 20223924 PMC2897704

[B62] Stuckenschneider T. Askew C. D. Menêses A. L. Baake R. Weber J. Schneider S. (2019). The effect of different exercise modes on domain-specific cognitive function in patients suffering from Parkinson’s disease: A systematic review of randomized controlled trials. *J. Parkinsons Dis.* 9 73–95. 10.3233/JPD-181484 30741688

[B63] van Wouwe N. C. Band G. P. H. Ridderinkhof K. R. (2011). Positive affect modulates flexibility and evaluative control. *J. Cogn. Neurosci.* 23 524–539. 10.1162/jocn.2009.21380 19925199

[B64] Verbruggen F. Logan G. D. (2009). Proactive Adjustments of response strategies in the stop-signal paradigm. *J. Exp. Psychol. Hum. Percept. Perform.* 35 835–854. 10.1037/a0012726 19485695 PMC2690716

[B65] Voelcker-Rehage C. Godde B. Staudinger U. M. (2011). Cardiovascular and coordination training differentially improve cognitive performance and neural processing in older adults. *Front. Hum. Neurosci.* 5:26. 10.3389/fnhum.2011.00026 21441997 PMC3062100

[B66] Wang T. Xiao G. Li Z. Jie K. Shen M. Jiang Y. (2020). Wuqinxi exercise improves hand dexterity in patients with Parkinson’s disease. *Evid. Based Complement. Alternat. Med.* 2020:8352176. 10.1155/2020/8352176 33178323 PMC7644302

[B67] Wiesner C. Vliet V. van Butt E. Pavensta H. Sto M. Linder S. (2008). Changes in gray matter induced by learning—revisited. *PLoS One* 3 1–5. 10.1371/journal.pone.0002669 18648501 PMC2447176

[B68] Wykes T. Brammer M. Mellers J. Bray P. Reeder C. Williams C. (2002). Effects on the brain of a psychological treatment: Cognitive remediation therapy. *Br. J. Psychiatry* 181 144–152. 10.1017/s0007125000161872 12151286

[B69] Wylie S. A. Richard Ridderinkhof K. Elias W. J. Frysinger R. C. Bashore T. R. Downs K. E. (2010). Subthalamic nucleus stimulation influences expression and suppression of impulsive behaviour in Parkinson’s disease. *Brain* 133 3611–3624. 10.1093/brain/awq239 20861152 PMC2995881

[B70] Xiao-Xi Z. (2012). Aberrant plasticity and “learned” motor inhibition in Parkinson’s disease. *Acta Physiol. Sin.* 64 543–549. 23090495

[B71] Xu L. Tang D.-D. Chen A.-T. (2013). The mechanisms and influential factors of the tradeoff between proactive and reactive cognitive control. *Adv. Psychol. Sci.* 20 1012–1022. 10.3724/sp.j.1042.2012.01012

[B72] Yang Y. Li X. Y. Gong L. Zhu Y. L. Hao Y. L. (2014). Tai chi for improvement of motor function, balance and gait in Parkinson’s disease: A systematic review and meta-analysis. *PLoS One* 9:e102942. 10.1371/journal.pone.0102942 25047456 PMC4105461

[B73] Ye Z. Altena E. Nombela C. Housden C. R. Maxwell H. Rittman T. (2014). Selective serotonin reuptake inhibition modulates response inhibition in Parkinson’s disease. *Brain* 137 1145–1155. 10.1093/brain/awu032 24578545 PMC3959561

[B74] Zandbelt B. B. van Buuren M. Kahn R. S. Vink M. (2011). Reduced proactive inhibition in schizophrenia is related to corticostriatal dysfunction and poor working memory. *Biol. Psychiatry* 70 1151–1158. 10.1016/j.biopsych.2011.07.028 21903198

[B75] Zhang F. Bai Y. H. Zhang J. (2014). The influence of “wuqinxi” exercises on the lumbosacral multifidus. *J. Phys. Ther. Sci.* 26 881–884. 10.1589/jpts.26.881 25013288 PMC4085213

[B76] Zou L. Sasaki J. E. Zeng N. Wang C. Sun L. (2018). A Systematic review with meta-analysis of mindful exercises on rehabilitative outcomes among poststroke patients. *Am. Congr. Rehabil. Med.* 99, 1–8. 10.1016/j.apmr.2018.04.010 29738744

